# Supplementary material for: The Secondary Use of Data to Support Medication Safety in the Hospital Setting: A Systematic Review and Narrative Synthesis
Source: Pharmacy (Basel). 2021 Dec 13;9(4):198. doi: 10.3390/pharmacy9040198 (PMC8706071; doi:10.3390/pharmacy9040198)
Supplement: Supplementary file 1 [file pharmacy-09-00198-s001.zip › pharmacy-1456028-supplementary.pdf]

pharmacy

MDPI

Supplementary file S1:

The search facets used across all four databases and an example search strategy used within EMBASE.

Table S1. The facets used for search strategy and its justification. The population, intervention, and outcomes have been underlined in the table below.

Number assigned to each facet:	Facet term used:	Boolean term:	Justification for facet term used:
1	Electronic data systems/surveillance	(1 AND 2 AND 3) OR 4	This literature review aims to look at secondary use of data from hospital electronic prescribing and pharmacy systems, as interventions.
2	Medication safety		Medication errors and/or adverse drug events are the outcome measure(s) for the interventions specified above. In order to capture the literature relating to medication errors, wider terms relating to medication safety such as adverse drug events were also used.
3	(Quality and safety) or (Hospital)		The setting (or population) specified were hospitals and all terms relevant to hospitals were therefore included. This PICO format helps answer our first 3 objectives stated above, with hospital as a setting. Some papers which focused on secondary use of data for quality improvement in relation to medication safety in healthcare did not specifically state the term hospital(s) in the title and abstract (as a keyword) or have it as a MeSH term. Therefore the following facet combination: hospital and (electronic data and surveillance) and medication safety would not be sensitive enough to retrieve all the relevant papers. So the terms for quality and safety were merged with the facet hospital with the Boolean term 'or' to ensure the search is sensitive enough to retrieve these papers. This helped us explore a wider range of literature focusing on secondary use of electronic data for quality improvement in healthcare. Therefore all terms relating to quality and safety or hospital were combined in this facet to produce a wider range of relevant literature.
4	'Secondary adj4 data'		This keyword was added with an 'or' Boolean term to bring up all papers sensitive to secondary use of data which may not have been included with [(quality and safety) or hospital] facet but may explore themes regarding secondary use of data.

The search terms used for EMBASE:

EMBASE subject headings (MeSH) and keywords:

1. electronic prescribing/

2. computerized provider order entry/
3. Electronic medical record/
4. *hospital information system/
5. *Decision support system/
6. (medical audit/ or feedback system/) and (information system/)
7. computer assisted drug therapy/
8. Secondary* data*
9. Secondary* use*
10. Secondary* adj4 data*
11. secondary* adj3 EHR*
12. secondary* adj3 electronic* health* record*
13. Surveillance* adj3 information* system*
14. ((computer* or electronic*) adj3 (detect* or surveillance*))
15. Medication* adj1 monitor*
16. Medicine* adj1 monitor*
17. Technovigilance*
18. Computer* adj4 surveillance*
19. Computer* adj4 monitor*
20. Electronic* prescri*
21. E-Prescri*
22. ePrescri*
23. prescri* adj3 data*
24. Decision* support* system*
25. Computer* prescri* support* system*
26. Computer* adj3 prescri*
27. electronic* adj3 prescri*
28. Automat* prescri*
29. Prescri* automat* screen* system*
30. Computer* information*
31. Computer* adj4 Adverse* drug*
32. Computer* adj4 patient* information*
33. Computer* physician* decision* support*
34. CPOE
35. Computer* physician* order*
36. physician* order* entry*
37. Computer* provider* order*
38. provider* order* entry*
39. Computer* prescriber* order*
40. prescriber* order* entry*
41. Computer* prescription* order*

42. prescription* order* entry*
43. Medication* order* entry*
44. Medicine* order* entry*
45. drug*order* entry*
46. dispen* adj2 data*
47. Hospital* dispen*
48. Pharmacy* adj2 dispen*
49. Clinic* adj2 information* system*
50. computer* pharmacy* record*
51. Electronic* medication* adj1 administration* record*
52. Electronic* medication* adj1 administration* system*
53. Electronic* drug* adj1 administration* record*
54. Electronic* drug* adj1 administration* system*
55. administration* data*
56. Medication* administration* system*
57. EPMA*
58. EMAR*
59. hospital* computer* program*
60. hospital* information* system*
61. hospital* data*
62. electronic* health* record*
63. electronic* clinical* system*
64. pharmacy* computer* system*
65. (Electronic* or computer*) adj2 discharg*
66. ((electronic* database*) and (clinical* data*))
67. ((pharmacy* or medication* or medicine*) adj1 (system*or data*) and (monitor* or access* or assess* or surveillance* or vigilance* or collect* or review* or identif* or analys* or examin* or investigat* or intervention* or compare*))
68. 1 or 2 or 3…. or 68
69. overdose/di, dt, pc [Diagnosis, Drug Therapy, Prevention]
70. drug toxicity/di, dt, pc [Diagnosis, Drug Therapy, Prevention]
71. medication error/
72. inappropriate prescribing/
73. Prescribing patterns
74. *treatment failure/
75. *Treatment errors
76. Adverse drug events
77. *drug administration/
78. Medication* adj2 error*
79. medicine* adj2 error*

80. drug* adj2 error*
81. prescri* adj2 error*
82. Dos* adj2 error*
83. Dos* adj2 mistake*
84. prescri* adj2 mistake*
85. Incorrect* adj1 prescri*
86. wrong* adj2 prescri*
87. inappropriate* adj1 prescri*
88. prescri* practice*
89. Duplicate* prescri*
90. Transcri* error*
91. Medication* adj2 mistake*
92. medicine* adj2 mistake*
93. drug* adj2 mistake*
94. prescri* adj2 mistake *
95. Medication* related* problem*
96. medicine* related* problem*
97. drug* related* problem*
98. Medication* related* event*
99. medicine* related* event*
100. drug* related* event*
101. Adverse* drug* event*
102. clinical* incident*
103. adverse* clinical* event*
104. Incorrect* dos*
105. wrong* dos*
106. inappropriate* dos*
107. medication* adj2 safe*
108. medicine* adj2 safe*
109. miss* adj2 medicine*
110. miss* adj2 medication*
111. miss* adj2 drug*
112. miss* adj2 dos*
113. omitted* adj2 medicine*
114. omitted* adj2 medication*
115. omitted* adj2 drug*
116. omitted* adj2 dos*
117. overdue* adj2 medicine*
118. overdue* adj2 medication*
119. overdue* adj2 drug*

120. overdue* adj2 dos*
121. delay* adj2 medicine*
122. delay* adj2 medication*
123. delay* adj2 drug*
124. delay* adj2 dos*
125. overdose* adj2 medicine*
126. overdose* adj2 medication*
127. overdose* adj2 drug*
128. underdos* adj2 medicine*
129. underdos* adj2 medication*
130. underdos* adj2 drug
131. administration* error*
132. appropriate* prescri*
133. Appropriate* treatment*
134. clinical* quality*
135. Dispens* error*
136. Dispens* mistake*
137. Drug* mishap*
138. Medication* mishap*
139. Wrong* drug*
140. Incorrect* drug*
141. Medication* adj2 discrepanc*
142. Medicine* adj2 discrepanc*
143. drug* adj2 discrepanc*
144. **<u>69 or 70 or 71…. or 143</u>**
145. *quality of healthcare /
146. *Patient safety/
147. Quality Improvement/
148. *Risk management/
149. *Risk assessment/
150. quality* adj2 safety* measurement*
151. quality* adj2 healthcare*
152. quality* adj2 health* care*
153. quality* improvement*
154. patient* safety*
155. (improv* quality* adj2 (efficiency* or care* or safety*))
156. improv* care*
157. Prevent* adj2 harm*
158. Reduc* adj2 harm*
159. Safety* adj1 Improv*

160. 145 or 146 or 147… or 159
161. Hospital units/
162. secondary health care/
163. tertiary health care/
164. Transfer discharge/
165. inpatients/
166. Hospital*
167. Tertiary* care*
168. Secondary* care*
169. acute* care*
170. Inpatient*
171. ward*
172. emergency* department*
173. secondary* healthcare*
174. tertiary* healthcare*
175. intensive* care* unit*
176. ICU
177. ITU
178. critical* care* unit*
179. 161 or 162 or 163… or 178
180. Secondary* use* adj4 data*
181. [68 and 144 and (160 or 179)] or 180

Table S2. Key summary of the above search strategy used in EMBASE.

Number:	Which facet each number represents:
68	Electronic systems (prescribing and pharmacy hospital systems)
144	Medication safety
160	Quality or safety
179	Hospital
180	Secondary* use* adj4 data*
Final combination for search	[Electronic system **and** medication safety **and** (quality or safety or hospital)] **or** secondary* use* adj4 data*

Supplementary file S2:

Exclusion criteria for title screening

Table S3. Title exclusion list: (If any of the factors listed below were mentioned with computerised prescribing order entry, electronic prescribing, electronic hospital pharmacy system, or any other electronic prescribing system then the paper was included at the title screening stage).

	Exclusion:
**Electronic systems:**	1. Paper based prescribing or non-technological system used
	2. Any database other than prescribing, pharmacy, or drug administration systems, clinical laboratory database
	3. Industrial post marketing drug surveillance

	4. Incident reporting system alone
	5. Computerised trigger tools, reminders or risk predictors alone
	6. Information systems that are used by non-healthcare professionals e.g. Consumer based technology
	7. Automated storage, automated medication dispensing system (in non-pharmacy based setting e.g. wards) medication cassettes, medication carts or distribution cabinets
	8. Unit dose systems
	9. Bar code technology
	10. Smart pumps
	11. Blood transfusion databases
Medication safety:	12. Adverse drug reactions alone
	13. Non medication or electronic database related papers e.g. looking solely at prevalence studies of diseases, patient costs, pharmacokinetic or pharmacodynamics studies, non-prescribed medication studies.
	14. Policy based papers regarding disciplinary action post medication errors
Setting:	15. Any non-hospital based setting alone e.g. primary care, state agencies, clinical registries, clinical trials of drugs etc.
Other:	16. Legal or ethical issues regarding deceased patient data
	17. Healthcare Failure Mode and Effect Analysis Studies

Abstract screening criteria

1. **'Secondary use of electronic prescribing/pharmacy data'**: the reuse of clinical and/or operational data from an EP or EHP system for purposes other than direct patient care or the original purpose for which the data was used.
2. **'Intervention based on secondary use of electronic prescribing/pharmacy data'**: The reuse of the data from an electronic prescribing or pharmacy system for secondary purpose(s) with the intent of changing or improving a process either alone or in combination with other intervention(s). The actual implementation of an electronic system was not considered an intervention. Examples can be seen below in Table S3.

Table S4. examples of 'secondary use of data' interventions.

Secondary use of data intervention used to:	Example:
Alter or improve another intervention	Existing electronic prescribing data is used to identify and then evaluate an intervention. Prescribing trend in data is assessed and causes alterations to be made to the policy for prescribing. Prescribing data is checked again and there is an increase in policy adherence.<br><br>Data is being re-used to assess the current situation and influences change within the practice/organisation leading to change i.e. data is driving the improvement.
Combined with another intervention	Existing electronic prescribing data is extracted and fed back to clinicians to improve their prescribing habits.<br><br>Data is being re-used to improve medication safety in combination with another intervention i.e. feedback with the aim of driving the improvement.

Table S5. Abstract screening tool. Exclude all abstracts that look at any of the criteria listed in the title exclusion criteria list and follow the inclusion criteria in table two under the title 'formal criteria' with the guidance of the extra notes.

Category	Formal criteria	Extra notes
1. Empirical studies on 'secondary use of data' intervention	1a. Paper focuses on medication safety, quality, or medication errors,<br><br>AND<br>1b. Paper describes a 'secondary use of data' intervention ,<br><br>AND<br>1c. The paper reports on re-use of electronic data from the following data sources: electronic prescribing or electronic pharmacy systems in one or more hospital(s),	• Every time the phrase 'secondary use of data' or 'secondary use of data intervention' is used then please refer back to the definitions stated above.<br>• A systematic review is not an empirical study hence cannot be allocated to category 1 and as it does quantify medication errors using data it cannot be allocated to category 3. However if the systematic review discusses the 'secondary use of data' intervention or concept then it can be allocated to category 2.<br>• If the paper does not specify whether the data is collected from the hospital electronic prescribing system or electronic pharmacy system but states a hospital electronic system then this should still be included if the data type for the outcome measure is stated and is medication safety related. E.g. if the medication error rates for certain drugs are stated then this information can only be collected by the pharmacy system or prescribing system.
2. Non-intervention based evaluation of 'secondary use of data'	Electronic secondary use of data concept/process is discussed in relation to healthcare in general relating to quality improvement.	• I.e. 'evaluative' without the empirical component e.g. systematic reviews.<br>• These may discuss an intervention but not implement it.
3. Non-intervention based study of medication quality/safety which makes 'secondary use' of pharmacy data	The paper does not include a intervention but uses existing electronic hospital data (from pharmacy and prescribing systems) to quantify the following topic areas:<br>a. Medication safety/quality<br>b. Medication errors	• This category is for papers which use existing electronic data from electronic prescribing or pharmacy hospital systems. This data is used to quantify topic areas listed in the criteria. However, there is no evaluation or intervention and the purpose of the papers is to identify frequencies of the measures listed.

Category 3 was excluded from the systematic literature review.

Table S5 can be used in conjunction with Figure S1 below.

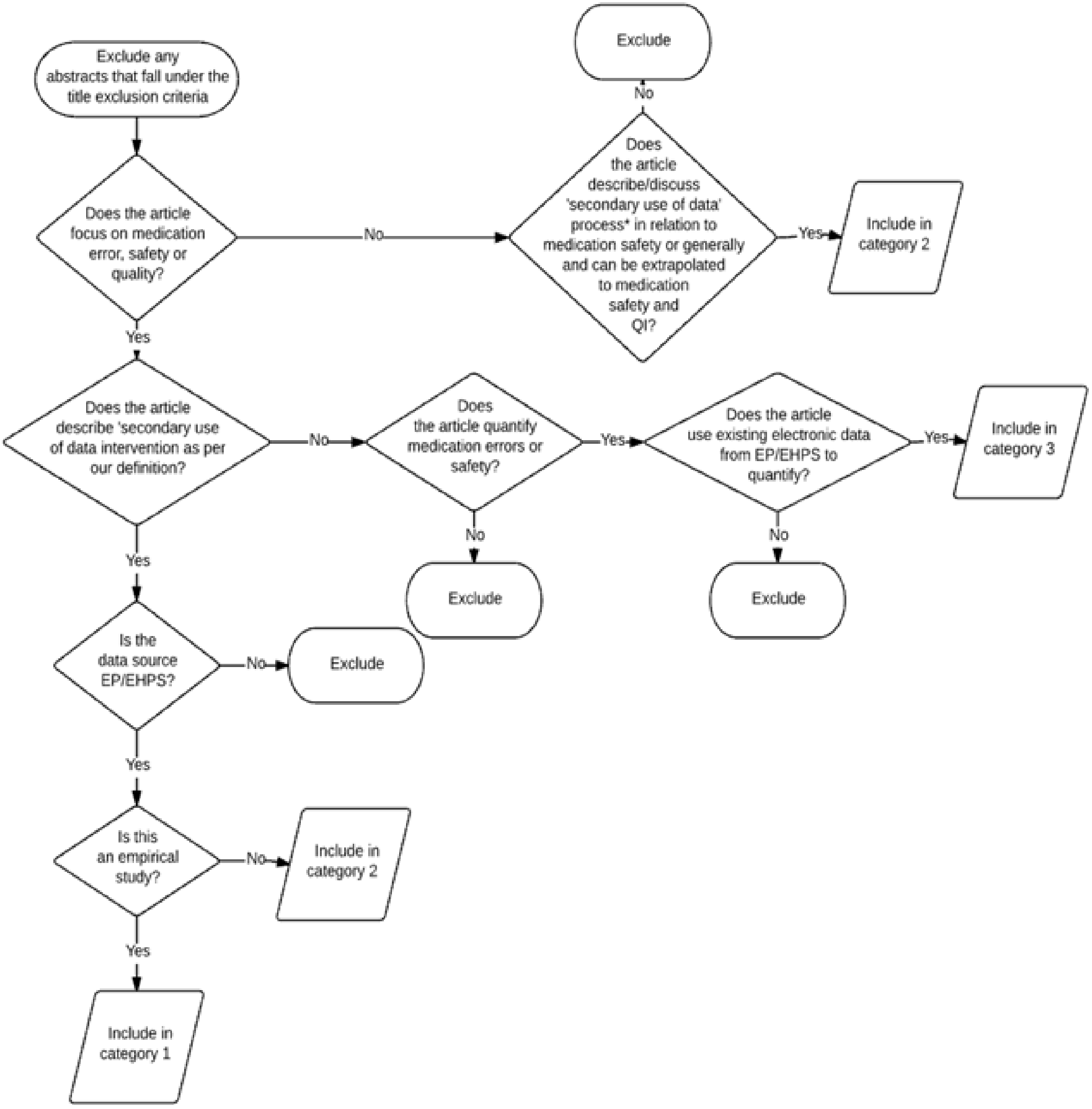

Figure S1. Abstract screening tool – flow diagram.

*Abbreviation:

EP – Electronic prescribing system

EHPS: Electronic hospital prescribing system

Full text screening criteria for including articles

1. **'Secondary use of electronic prescribing/pharmacy data'**: the reuse of clinical and/or operational data from an EP or EHP system for purposes other than direct patient care or the original purpose for which the data was used.
2. **'Intervention based on secondary use of electronic prescribing/pharmacy data'**: The reuse of the data from an electronic prescribing or pharmacy system for secondary purpose(s) with the intent of changing or improving a process either alone or in combination with other intervention(s). The actual implementation of an electronic system was not considered an intervention.

The definition for 'secondary use of electronic pharmacy/prescribing data' excludes the following: the use of existing data to evaluate another intervention, or just using data to determine the frequency or quantity of the outcome measures.

Table S6. Full text inclusion criteria: Include paper in category 1 if all 3 criteria are met:

Criteria number:	Inclusion criteria:
1.	Empirical study in one or more hospital(s) - with 'secondary use of data intervention'
2.	Existing electronic data used from electroic prescribing and/or electronic hospital pharmacy system, please see figure 1 below for full detail
3.	Study focuses on medication safety, medication errors or quality improvement in medication

For the second category of articles a flowchart was used to determine whether articles would be included or excluded, please see figure S2 below. Articles were only included in 2a if they could be applied to healthcare in relation to medication safety.

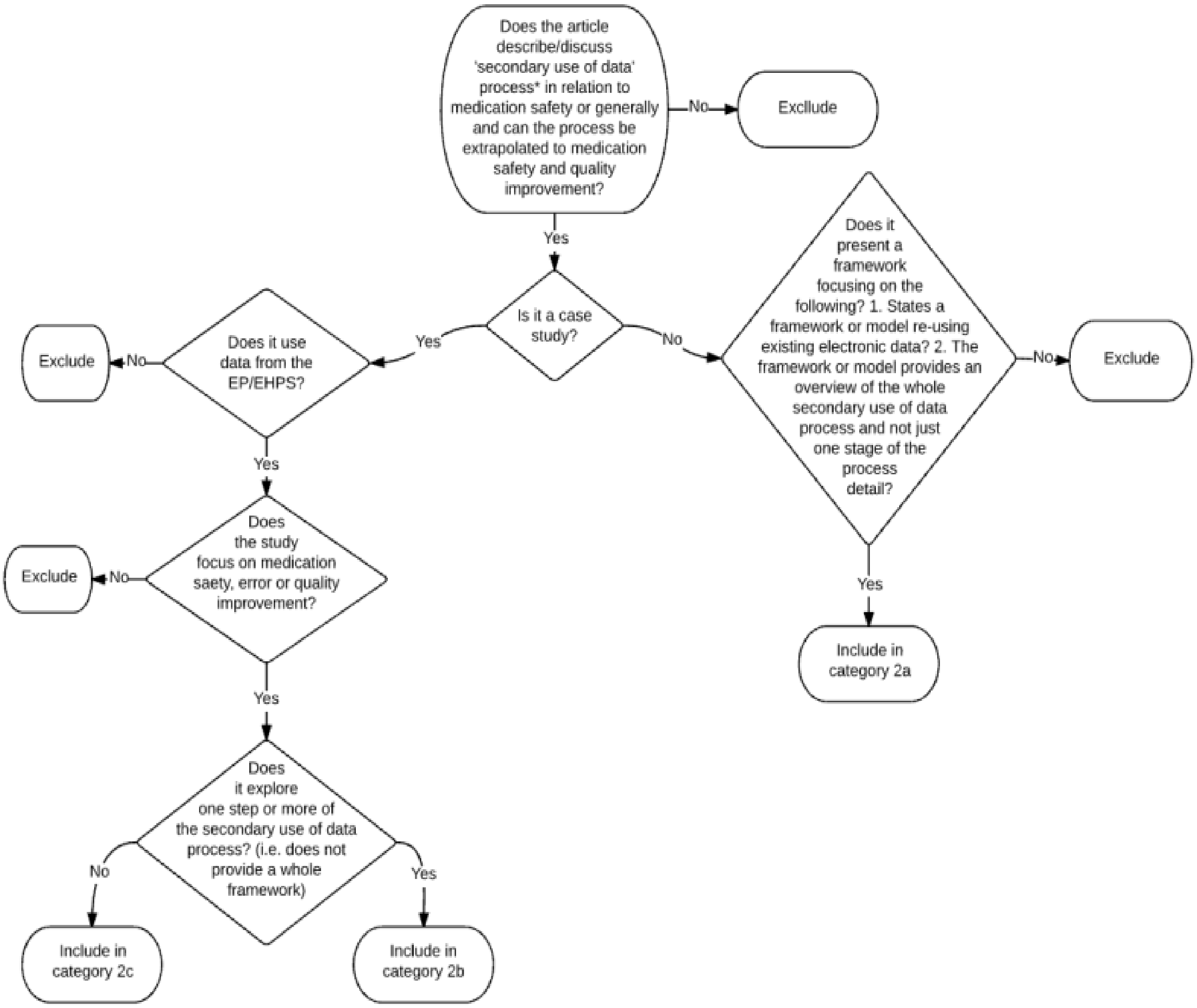

Figure S2. for category 2: Inclusion criteria for category 2 articles.

Key: EP – electronic prescribing， EHPS – electronic hospital pharmacy system， Category 2a and 2b was kept and the other categories were excluded from the review

Supplementary file S3:

Data extraction

**Category:**	**Information extracted from each paper:**
1	Author
	Title
	Country
	Type of hospital (if specified)
	Aim
	Team involved, clinical setting and end users
	Electronic system used (type and brand if specified)
	Study evaluation design
	Type of data used from electronic system(s)
	Other interventions linked with secondary data use
	Outcome measure(s)
	Limitation(s) of study
	Barrier(s), limitation(s) and/or driver(s) of secondary use of data stated
	Contextual factor(s) considered
2	Author
	Title
	Country
	Study design
	Framework proposed
	Data type used to produce framework
	Framework evaluated

For table S6 the following details were extracted from articles that implemented a feedback intervention:

- Author
- Characteristics of feedback:
  - Data is valid based on the receivers perspective
  - Credibility of data in organisation (for receivers to believe the data presented)
  - Source and timeliness of data
  - Benchmarking (comparing results amongst others and promoting healthy competition)
  - Leaders presenting data (trusted individuals presenting data)
  - Data is individualised (personalised data)
  - Constant feedback (not a one off account of feedback)

Supplementary file S4:

A table presenting data from the studies which were not intervention based literature discussing the different factors to be considered for secondary use of data.

Table S7. Studies providing an overview of secondary use of data frameworks in criterion two.

**Author(s)**	**Title**	**Country**	**Study design:**	**Framework presented:**

Bain, M.R.S., et al. 1997 (5)	Routinely collected data in national and regional databases – an underused resource	UK	Narrative literature review	Consists of 6 main stages to consider:<br>1. Identification of useful data routinely entered<br>o What databases are available<br>o What data is available on these databases<br>o Can the data be used in an innovative manner<br>2. Confidentiality/privacy<br>o The approval required to access confidential information if this information is needed<br>o potential privacy issue when trying to link information<br>o Need patient consent in certain circumstances<br>3. Data quality<br>o Data completeness<br>o Data accuracy and validity<br>o Timeliness of data<br>o Factors to improve: information on data quality should be available and clinicians should improve the data entered into the systems (this not just the responsibility of the people who enter the data but also the people who produce the data). The cause of poor quality data is lack of education to healthcare professionals.<br>4. Appropriateness of the routine data for the selected purpose.<br>5. Analysis and interpretation of the data<br>o Can get data in two forms, one which has already been analysed or the other where you are required to conduct the analysis yourself. Need for correct equipment and knowledge in order to conduct the latter step.<br>o Specialist help maybe required where necessary such as statisticians<br>o Need to ensure the data used is in fact representing the truth and is not biased, therefore need to be aware of the weaknesses and strengths of the data (so need to consider coding, data collection method and definitions used).<br>o Need to consider the countries in which the data was collected which can influence the data factors listed above.<br>6. Ethical issues<br>o Present data in the clearest and honest form possible with its limitations and prevent over interpretation.
Danciu, I., et al. 2014 (34)	Secondary use of clinical data: The Vanderbilt approach	USA	Reflective piece on a case study	9 factors to consider:<br>1. Need clinical enterprise need software to undergo data extraction e.g. StarPanel and EDW but both are limited and require specialist knowledge and input.<br>2. Data identification: this can be done using different codes e.g. Med-Ex for medication and stored in RD.<br>3. De-identification of data and storage: removal of personal information<br>4. Specialist skills and knowledge: required to keep repository updated. Knowledge of data re-identification risks, awareness of privacy and ethic and ensuring governance rules have been adhered too.<br>5. Presenting the need for this data being reused<br>6. Improving systems due to incentive of gaining access to reusing the database.<br>7. Translational use of clinical data envisaged and supported<br>8. Making access to data warehouse known and available to all at all level. Increasing the knowledge and use would increase the demand and reduce pressure on teams providing support; nevertheless support is still required from individuals to users when required. Self-service for data extraction could be promoted and if that is not possible then specialist help can be sought.

				9. Having a request process infrastructure which supports secondary use of data.<br>Need to be aware of the following:<br>o Incomplete data due to lack of time from primary system users<br>o Understand the data significance, whether the data is meaningful or not<br>o Understand the policy and privacy requirements<br>o Understand the when, how and purpose of data collection before reuse.
Galster, G., 2012 (35)	Why is clinical information not reused?	Denmark	Systematic literature review	4 major barriers identified resulting in lack of clinical data reuse:<br>1. Lack of data availability when required<br>2. The data source use is prohibited<br>3. Data cannot be used in the form available (reasons for this spilt in 3 categories technical, political and quality reasons)<br>4. Data is inadequate to be reused (2 main reasons insufficient reliability and inadequate relevance) .All placed under technical, organisational, legal and medical issues.
Holzer, K. and W. Gall 2011. (36)	Utilizing IHE-based Electronic Health Record Systems for Secondary use	Austria	Systematic literature review	Requirements for SUD from EHRs:<br>1. Factors to consider for secondary use of data: Security measures to be considered, data formatting, user groups for secondary use of data and their requirements and query formulation process.<br>2. System requirements (8 in total): standard terminology, cross patient/domain retrieval, selection of document, anonymisation, query within retrieved document, user roles, compliance with SUD policies and sensitisation within population.
Safran, C., et al. 2007 (33)	Toward a national framework for the secondary use of health data	USA	Qualitative work - discussion between experts.	5 recommendations:<br>1. Increase transparency of data use and promote public awareness, focus ongoing discussions on data access, use, and control not on ownership,<br>2. Discuss privacy policies and security for secondary use of health data increase public awareness of benefits and challenges associated with secondary use of health data,<br>3. Create taxonomy for secondary uses of health data,<br>4. Address comprehensively the difficult, evolving questions related to secondary use of health data and focus national and<br>5. State attention on the secondary use of health data.
